# Evidence for NADPH oxidase activation by GPR40 in pancreatic β-cells

**DOI:** 10.1080/13510002.2020.1757877

**Published:** 2020-05-01

**Authors:** Gabriela Nunes Marsiglio-Librais, Eloisa Aparecida Vilas-Boas, Christopher Carlein, Markus Daniel Alexander Hoffmann, Leticia Prates Roma, Angelo Rafael Carpinelli

**Affiliations:** a Department of Physiology and Biophysics, Institute of Biomedical Sciences, University of São Paulo (USP), São Paulo, Brazil; b Department of Biophysics, Center for Human and Molecular Biology, CIPMM, Saarland University, Homburg/Saar, Germany

**Keywords:** GPR40, NADPH oxidase, ROS, p47^phox^ translocation, BRIN-BD11, GW9508, Linoleic acid, NOX2 KO islets, roGFP2-Orp1, Insulin secretion

## Abstract

**Objective:** Investigate the involvement of the fatty acids receptor GPR40 in the assembly and activation of NADPH oxidase and the implications on pancreatic β-cell function.

**Methods:** BRIN-BD11 β-cells were exposed to GPR40 agonist (GW9508) or linoleic acid in different glucose concentrations. Superoxide and H_2_O_2_ were analyzed, respectively, by DHE fluorescence and by fluorescence of the H_2_O_2_ sensor, roGFP2-Orp1. Protein contents of p47^phox^ in plasma membrane and cytosol were analyzed by western blot. NADPH oxidase role was evaluated by p22^phox^ siRNA or by pharmacological inhibition with VAS2870. NOX2 KO islets were used to measure total cytosolic calcium and insulin secretion.

**Results:** GW9508 and linoleic acid increased superoxide and H_2_O_2_ contents at 5.6 and 8.3 mM of glucose. In addition, in 5.6 mM, but not at 16.7 mM of glucose, activation of GPR40 led to the translocation of p47^phox^ to the plasma membrane. Knockdown of p22^phox^ abolished the increase in superoxide after GW9508 and linoleic acid. No differences in insulin secretion were found between wild type and NOX2 KO islets treated with GW9508 or linoleic acid.

**Discussion:** We report for the first time that acute activation of GPR40 leads to NADPH oxidase activation in pancreatic β-cells, without impact on insulin secretion.

## Introduction

Although glucose is the main secretagogue of insulin, other molecules including hormones, amino acids and free fatty acids (FFAs) also modulate insulin secretion [1–4]. G protein-coupled receptors activated by FFAs, namely GPRs, were identified in different cell types [5]. Among them, GPR40 is highly expressed in pancreatic β-cells and has a great affinity for saturated and unsaturated long-chain FFAs. The activation of GPR40 triggers signaling cascades involved in the process of insulin secretion [6–8]. The complete picture following GPR40 activation is still unknown, but it involves activation of phospholipase C (PLC), via Gαq protein, leading to the hydrolysis of phosphatidylinositol 4,5-biphosphate (PIP_2_) in diacylglycerol (DAG) and inositol 1,4,5-triphosphate (IP_3_), which respectively activates protein kinase C (PKC) and mobilizes calcium from endoplasmic reticulum [7,9,10].

Importantly, it was shown that GPR40 inhibition impairs FFA-stimulated insulin secretion *in vitro* [1]. In addition, pancreatic islets of human donors with type 2 diabetes mellitus have diminished expression of GPR40 [11] and obese individuals have a higher frequency of GPR40 mutations, leading to impairment of insulin secretion [12].

Due to these findings, GPR40 was proposed as a valuable target for the development of new drugs for the treatment of type 2 diabetes. Thus, in the past years, several agonist molecules have been developed and used in *in vitro*, mouse models [13–18] and clinical trials [18–22] aiming to understand the impact of activating GPR40 for whole-body glycemic control.

Reactive oxygen species (ROS) have been suggested as signaling molecules important for insulin secretion, reviewed in [23]. Two important sources of ROS in pancreatic β-cells are the electron transport chain and the NADPH oxidases. NADPH oxidase produces superoxide and was originally described in phagocytic cells in which it is of great importance to eliminate bacteria and other pathogens [24,25]. The complex is composed by two membrane subunits, gp91^phox^ and p22^phox^, forming the flavocytochrome b558, catalytic core of the enzyme, the cytosolic subunits p67^phox^, p47^phox^ and p40^phox^, and the small GTPase Rac [24,26].

For activation of the complex, the organizer subunit p47^phox^ is phosphorylated by PKC, promoting the translocation of all cytosolic subunits to the membrane and their association with the membrane subunits. The assembled complex produces superoxide anions through the reduction of oxygen molecule, utilizing NADPH as an electron donor [24,26].

Despite its importance in phagocytes, NADPH oxidase is ubiquitously expressed [25]. Our group has previously demonstrated the expression of NADPH oxidase subunits in human and rodent pancreatic islets and insulin-secreting β-cell lines [27,28]. The superoxide produced may be involved in the process of insulin secretion, since the acute inhibition of NADPH oxidase by DPI or p47^phox^ oligonucleotide antisense promote the reduction of glucose-stimulated insulin secretion (GSIS) [29]. NADPH oxidase also participates in the palmitate-induced superoxide production and insulin secretion [30]. The mechanism, however, is still unclear.

On the other hand, NOX2 KO islets were shown to secrete more insulin upon glucose stimulation, and NOX2, therefore, would act as a negative modulator of insulin secretion, an effect attributed to modulation of cAMP [31,32]. In addition, inhibition of NADPH oxidase rescued the decreased glucose oxidation, elicited by oleate treatment. Thus, it is clear that much controversy exists regarding the role of NADPH oxidase-induced ROS on insulin secretion.

Considering the possible role of NADPH oxidase in the regulation of insulin secretion and that stimulation of GPR40 by FFAs leads to an increase in GSIS through PKC, which is a common pathway on the activation of NADPH oxidase; the aim of this work was to evaluate the involvement of GPR40 in the activation of NADPH oxidase and consequently the production of reactive oxygen species (ROS) production by pancreatic β-cells. Finally, we also evaluated insulin secretion and calcium homeostasis in NOX2 KO islets upon GPR40 activation. In this study, we used the agonist GW9508 and the unsaturated long-chain FFA linoleic acid, which are potent activators of GPR40 [13,33,34].

## MATERIALS and mEtHoDs

### Reagents

Reagents for SDS-PAGE were obtained from Bio-Rad (Richmond, CA, USA); dihydroethidium (DHE), RPMI 1640 culture medium, heat-inactivated fetal bovine serum, Opti-MEM medium, Lipofectamine 2000, RIPA lysis buffer and Fura-2 AM were obtained from Thermo Fischer Scientific (Waltham, MA, USA); Bradford Reagent and linoleic acid were obtained from Sigma-Aldrich (St. Louis, MO, USA); Insulin Ultra-Sensitive Assay kit was obtained from Cisbio (Codolet, France); GW9508 was obtained from Tocris Bioscience (Bristol, England, UK); VAS2870 was obtained from Enzo Life Sciences (Farmingdale, NY, USA); p22^phox^ siRNA (#sc-61892), control siRNA (#sc-37007), anti-p47^phox^ (#sc-14015; dilution 1:1000 in 5% BSA in TBST), anti-p22^phox^ (#sc-271968; dilution 1:500 in 5% BSA in TBST) and anti-CD73 (#sc-25603; dilution 1:1000 in 5% BSA in TBST) antibodies were obtained from Santa Cruz Biotechnology (Santa Cruz, CA, USA); goat anti-rabbit IgG HRP (#ab205718; dilution 1:10,000 in 3% skimmed milk in TBST) and goat anti-mouse IgG HRP (#ab97023; dilution 1:10,000 in 3% skimmed milk in TBST) secondary antibodies were obtained from Abcam (Cambridge, UK).

### Animals

C57BL/6J wild type (WT) and NOX2 deficient (KO) mice aged between 10 and 20 weeks were used for insulin secretion and Ca^2+^ measurements. All animals were maintained in the animal facility of the Center for Integrative Physiology and Molecular Medicine (CIPMM) of Saarland University devoid of murine pathogens. All procedures were in accordance with the ethical principles of the local ethical committee.

### Pancreatic islets isolation

After full inflation with 4 ml of collagenase P in Krebs Henseleit (KH) buffer (0.63 mg/ml), pancreas was removed and incubated for 25 min in the water bath at 37°C for digestion of the exocrine portion. Pancreas was then shaken manually, washed with KH buffer and centrifuged three times (1000 rpm / 5 min). Islets were collected in a stereomicroscope and cultured for 48 h before treatments.

### Cell lines

BRIN-BD11 cells were used for superoxide measurement and for the western blot of the subunit p47^phox^ in fractions from cytosol and membrane; roGFP2-Orp1 BRIN-BD11 cells were used for the assessment of H_2_O_2_ production. For superoxide measurement, cells were seeded at 2.5 × 10^5^ per well in 500 µl medium in a 24-well plate; for translocation of p47^phox^, cells were seeded at 1 × 10^7^ per flask in 10 ml medium in 75 cm^2^ tissue culture flasks; for H_2_O_2_ analysis, cells were seeded at 5 × 10^4^ cells per well in 200 µl medium in 96-well plates. Cells were allowed to adhere overnight and then exposed to different treatments.

### Culture of BRIN-BD11 and islets

BRIN-BD11 cells, roGFP2-Orp1 BRIN-BD11 cells and mice islets were cultured at 37°C in a humidified atmosphere containing 5% CO_2_ in complete RPMI 1640 medium containing 11.1 mM glucose and supplemented with 10% fetal bovine serum, 100 U/ml penicillin and 100 μg/ml streptomycin.

### Superoxide measurement

BRIN-BD11 cells were pre-incubated for 20 min in Krebs-Henseleit (KH) buffer supplemented with 11.1 mM glucose. After, the cells were incubated in KH buffer containing different glucose concentrations in the absence or presence of GW9508 or linoleic acid for 60 min. The fluorescence was then measured by the flow cytometer Guava EasyCyte (Merck Millipore – Billerica, MA, USA), using the DHE dye, as previously described [35].

### Translocation of p47^phox^ subunit to the membrane

To analyze the translocation of p47^phox^ from cytosol to the plasma membrane, cells were exposed to 20 μM GW9508 in KH buffer at 5.6 mM or 16.7 mM of glucose for different periods (0, 5, 10, 20, 30 and 40 min). After incubation, cells were disrupted using extraction buffer (10 mM Tris, 1 mM EDTA and 250 mM sucrose) containing protease and phosphatase inhibitors. Content was centrifuged for 15 min at 1000 g/4°C; pellet containing the nuclear fraction was discarded. Supernatant was collected and centrifuged at 12000g/4°C for 15 min. Supernatant containing the cytosolic fraction was collected and the pellet containing the membrane fraction was resuspended in extraction buffer. Protein concentration of each fraction was quantified by Bradford reagent and 40 μg of protein were submitted to SDS-PAGE and transferred to nitrocellulose membranes to be stained with specific antibodies against the protein of interest, p47^phox^, and the internal control, CD73.

### Small interfering RNA transfection

BRIN-BD11 cells were transfected for 7 h at 37°C in Opti-MEM medium containing Lipofectamine 2000 in the presence or absence of 40 nM of siRNA for p22^phox^ or scramble RNA as a negative control (Santa Cruz Biotechnology, Texas, USA). After transfection, BRIN-BD11 cells were maintained in complete RPMI 1640 medium containing 11.1 mM glucose for additional 48 h before analysis of knockdown by western blot.

### Western blot

Protein expression of p47^phox^ in membrane and cytosolic fractions and of p22^phox^ after transfection with siRNA were assessed by western blot. Total protein extracts (40 μg) were separated by SDS-PAGE and transferred to nitrocellulose membranes. Membranes were probed with specific antibodies against the proteins of interest, using anti-CD73 as internal control. Bands were quantified by densitometry using ImageJ Software.

### Hydrogen peroxide (H_2_O_2_) measurement

Measurements of intracellular levels of H_2_O_2_ were performed in roGFP2-Orp1 BRIN-BD11 cells using the CLARIOstar Microplate Reader (BMG LABTECH, Ortenberg, Germany). Temperature was set to 37°C and atmospheric conditions were set to 5% CO_2_ with the ventilation left open for O_2_ to diffuse freely in the system (18.8% O_2_). Protocol was divided into two steps: first in basal condition, followed by addition of different stimuli. Cytosolic roGFP emission was detected after excitation at 400 and 480 nm and emission at 500–530 nm. Fully oxidized (DA) and fully reduced (DTT) samples were prepared for the calculation of the degree of probe oxidation (OxD). The calculation of OxD values was done as previously described [36].

### Insulin secretion

After 1 h incubation with different conditions, islets from WT and NOX2 KO mice were checked for GSIS. Groups of 10 islets were collected to fresh tubes containing KH buffer with 0.5 mM glucose and incubated at 37°C for 45 min. Supernatant was discarded and substituted by KH buffer with either 5.6 or 8.3 mM of glucose and incubated at 37°C for 60 min. Supernatant was collected and insulin release was measured by Fluorescence Resonance Energy Transfer (FRET) using the Insulin Ultra-Sensitive Assay kit (Cisbio). The signal intensity was measured at 665 and 620 nm at the CLARIOstar Microplate Reader (BMG LABTECH).

### Ca^2+^ measurements

In order to assess total cytosolic Ca^2+^, islets from WT and NOX2 KO mice were loaded with 5 µM Fura-2 AM for 2 h in RPMI 1640 culture medium (incubated at 37°C and 5% CO_2_). In the last hour of loading, GW9508 (final concentration: 20 µM) or linoleic acid (final concentration: 30 μM) were added. Islets were then washed twice for 10 min in bicarbonate buffered KH solution with 0 mM glucose. After washing, the islets were placed under the microscope Axio Observer 7 (Zeiss, Oberkochen, Germany) and measurements were made every 2 s for 30 min. Protocol was divided into two steps: first in KH buffer without glucose, followed by addition of high glucose (20 mM). Islets were imaged using excitation at 340/380 nm and emission at 505 nm.

### Statistical analysis

Results are presented as means ± SEM. Statistical analysis was performed by One-way ANOVA followed by Dunnett, Sidak or Tukey or Two-way ANOVA followed by Tukey or Sidak, as appropriate and indicated in figure legends, using GraphPad Prism 7 software (La Jolla, CA, USA). The level of significance was considered for *p*<0.05.

## Results

### Effect of 1-hour exposure to GW9508 or linoleic acid on ROS production of BRIN-BD11 cells

**Figure 1.**
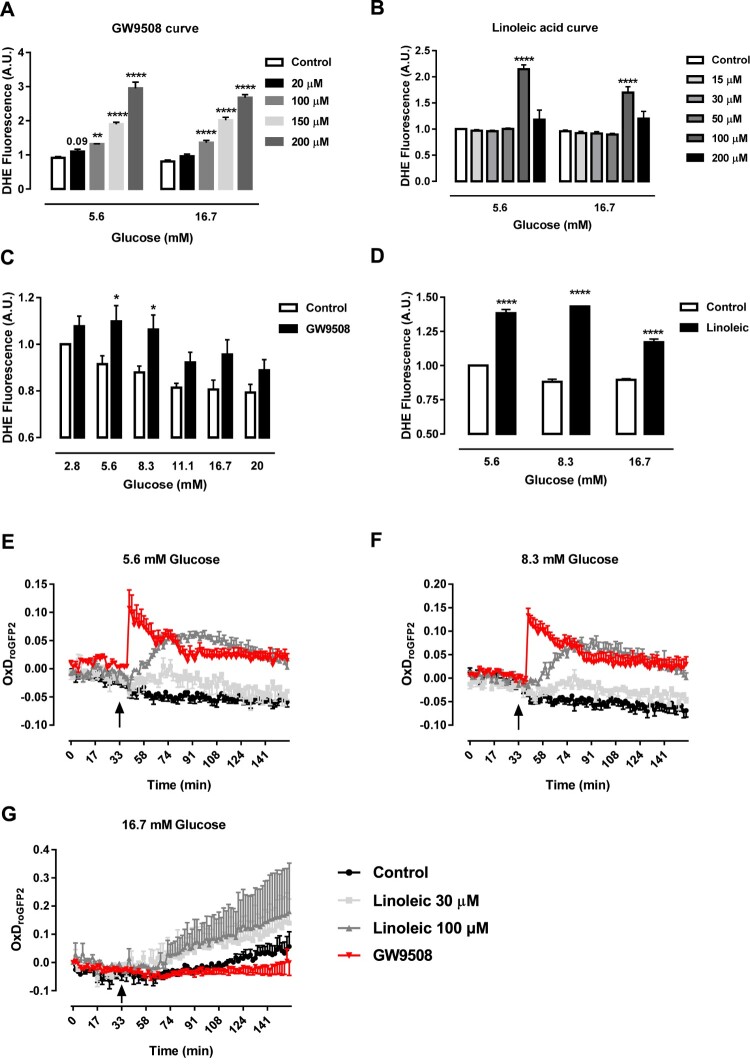
GW9508 and linoleic acid stimulate ROS production. (A) Production of superoxide by BRIN-BD11 cells incubated in the absence (Control) or presence of different concentrations of GPR40 agonist, GW9508, (20, 100, 150 or 200 μM) in 5.6 or 16.7 mM glucose for 60 min. Results are expressed as mean ± SEM of 3–8 independent experiments. **P<0.01 and ****P<0.0001 versus Control in corresponding glucose concentration. Two-way ANOVA followed by Dunnett. (B) Production of superoxide by BRIN-BD11 incubated in the absence (Control) or presence of different concentrations of linoleic acid (15, 30, 50, 100 or 200 μM) in 5.6 or 16.7 mM glucose for 60 min. Results are expressed as mean ± SEM of 4 independent experiments. ****P<0.0001 versus Control in corresponding glucose concentration. Two-way ANOVA followed by Dunnett. (C) Production of superoxide by BRIN-BD11 cells incubated in the absence (Control) or presence of 20 μM GW9508 in different glucose concentrations (2.8, 5.6, 8.3, 11.1, 16.7 and 20 mM) for 60 min. Results are expressed as mean ± SEM of 7–8 independent experiments. *P<0.05 versus Control in corresponding glucose concentration. Two-way ANOVA followed by Sidak. (D) Production of superoxide by BRIN-BD11 cells incubated in the absence (Control) or presence of 100 μM linoleic acid (Linoleic) in different glucose concentrations (5.6, 8.3, and 16.7 mM) for 60 min. Results are expressed as mean ± SEM of 4 independent experiments. ****P<0.0001 versus Control in corresponding glucose concentration. Two-way ANOVA followed by Sidak. (A–D) Production of superoxide was analyzed by flow cytometry using DHE dye. (E–G) Dynamic production of H_2_O_2_ by BRIN-BD11 roGFP2-Orp1 cells. Arrows indicate moment of addition of different conditions. Conditions: Control, 20 μM GPR40 agonist GW9508 (GW9508), 30 μM linoleic acid and 100 μM linoleic acid in different glucose concentrations: 5.6 mM (E), 8.3 mM (F) and 16.7 mM (G). Results are expressed as OxD_roGFP2_ of 3–6 independent experiments.

We first analyzed the effect of different concentrations of the GPR40 agonist GW9508 (20, 100, 150 and 200 μM) on the primary product of NADPH oxidase activity, superoxide, after 1-hour exposure of BRIN-BD11 cells in low (5.6 mM) and high (16.7 mM) glucose concentrations (Figure 1(A). Because GW9508 and the unsaturated FFA linoleic acid have similar binding sites to GPR40 [34], we also checked for superoxide production of BRIN-BD11 cells after 1-hour exposure to different concentrations of linoleic acid (15, 30, 50, 100 and 200 μM) in both 5.6 and 16.7 mM glucose (Figure 1(B)). Acute exposure to GW9508 promoted an increase in the total superoxide content in 5.6 mM of glucose in all concentrations tested, while in 16.7 mM glucose, the increase was seen only in 100, 150 and 200 μM (Figure 1(A)). Linoleic acid had no effect on superoxide production in any concentration tested, except for 100 μM in both 5.6 and 16.7 mM glucose (Figure 1(B)).

For next experiments, we chose 20 μM GW9508 and 100 μM linoleic acid. We then evaluated 1-hour production of superoxide in different glucose concentrations (Figure 1(C,D)). GW9508 led to increase of superoxide in 5.6 and 8.3 mM glucose and not in higher concentrations of glucose (Figure 1(C)). On the other hand, linoleic acid led to increase in superoxide in all concentrations of glucose tested (Figure 1(D)). This may be due to the fact that linoleic acid also acts through other pathways than the activation of GPR40.

As superoxide is rapidly converted into H_2_O_2_ by superoxide dismutases, we next checked for the production of H_2_O_2_ in the recently-generated BRIN-BD11 cells expressing a specific sensor for H_2_O_2_ in the cytosol (roGFP2-Orp1 BRIN-BD11) during 1-hour exposure to GW9508 (20 μM) or linoleic acid (30 or 100 μM) in different glucose concentrations: 5.6 mM (Figure 1(E)), 8.3 mM (Figure 1(F)) and 16.7 mM (Figure 1(G)). In 5.6 and 8.3 mM of glucose, there was an immediate increase in cytosolic H_2_O_2_ production right after the addition (arrow) of GW9508 and a more gradual increase after addition of 100 μM linoleic acid and only a slight increase with 30 μM linoleic acid (Figure 1(E,F)). In contrast to that, the addition of GW9508 did not lead to increase of the cytosolic H_2_O_2_ in 16.7 mM glucose concentration (Figure 1(G)).

### Effect of 1-hour exposure to GW9508 on the translocation of p47^phox^ from cytosol to membrane

**Figure 2.**
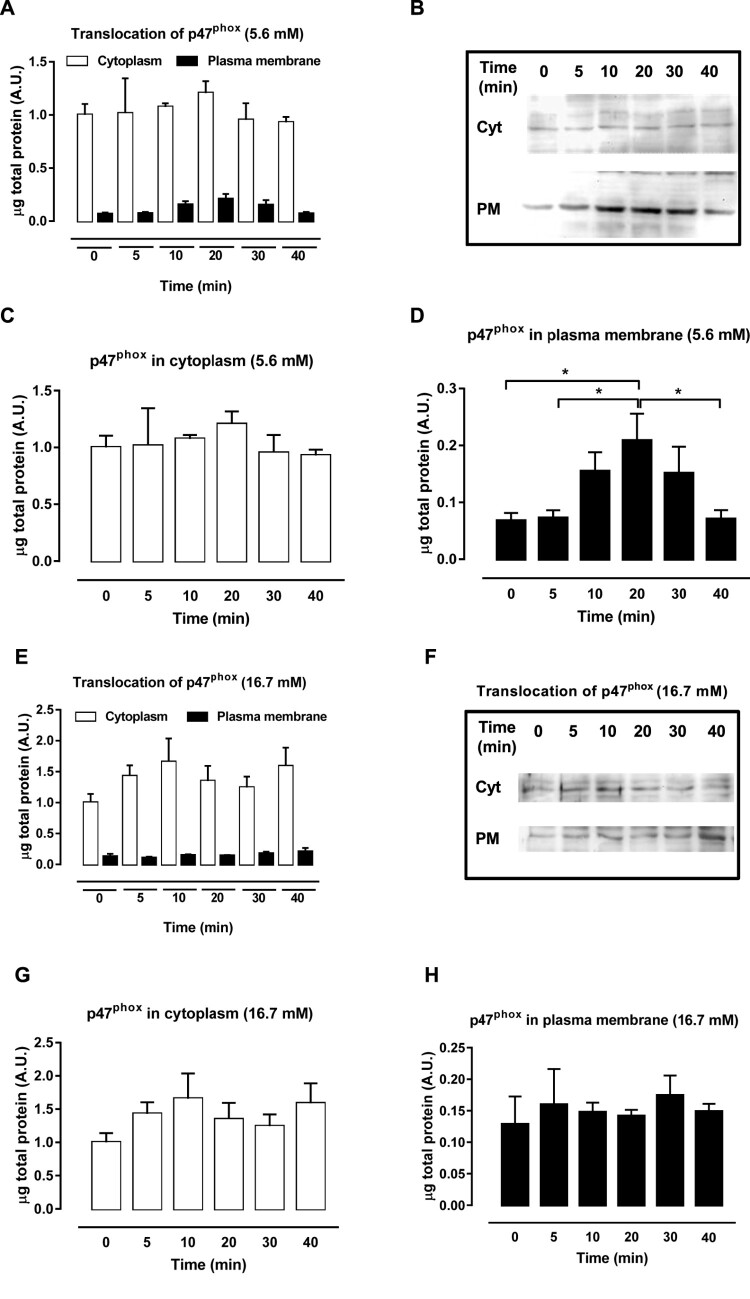
p47^phox^ migration from cytosol to plasma membrane after exposure to GW9508. (A–B) Protein expression of cytosolic and membrane fractions of p47^phox^ in BRIN-BD11 cells after GPR40 stimulation with 20 μM of GW9508 for 0, 5, 10, 20, 30 and 40 min in 5.6 mM glucose. (C) Cytosolic fractions separately. (D) Membrane fractions separately. Results are expressed as mean ± SEM of 4 independent experiments. *P<0.05. One-way ANOVA followed by Tukey. (E–F) Protein expression of cytosolic and membrane fractions of p47^phox^ in BRIN-BD11 cells after GPR40 stimulation with 20 μM of GW9508 for 0, 5, 10, 20, 30 and 40 min in 16.7 mM glucose. (G) Cytosolic fractions separately. (H) Membrane fractions separately. Results are expressed as mean ± SEM of 4 independent experiments.

The activation of the NADPH oxidase complex starts with the phosphorylation of p47^phox^, which promotes the migration of the cytosolic subunits to the plasma membrane and their association with membrane subunits. Therefore, to address whether NADPH oxidase was activated, the effect of the GPR40 agonist GW9508 on the translocation of the organizer subunit p47^phox^ from cytosol to the plasma membrane was analyzed after exposure of BRIN-BD11 cells to 20 µM GW9508 in different periods (0–40 min). For this experiment, we chose 5.6 mM (Figure 2(A–D)) and 16.7 mM (Figure 2(E–H)) of glucose. Analysis of cytosolic and membrane fractions was performed. In 5.6 mM glucose the organizer subunit p47^phox^ translocated from the cytosol (Figure 2(C)) to plasma membrane (Figure 2(D)) after 10–30 min of exposure to GW9508. No translocation was observed in 16.7 mM glucose (Figure 2(E–H)). This result shows that GPR40 activation with agonist GW9508 leads to assembly of the NADPH oxidase complex in low glucose.

### Effect of p22^phox^ knockdown or pharmacological inhibition of NADPH oxidase on the superoxide content after exposure to GW9508 or linoleic acid

**Figure 3.**
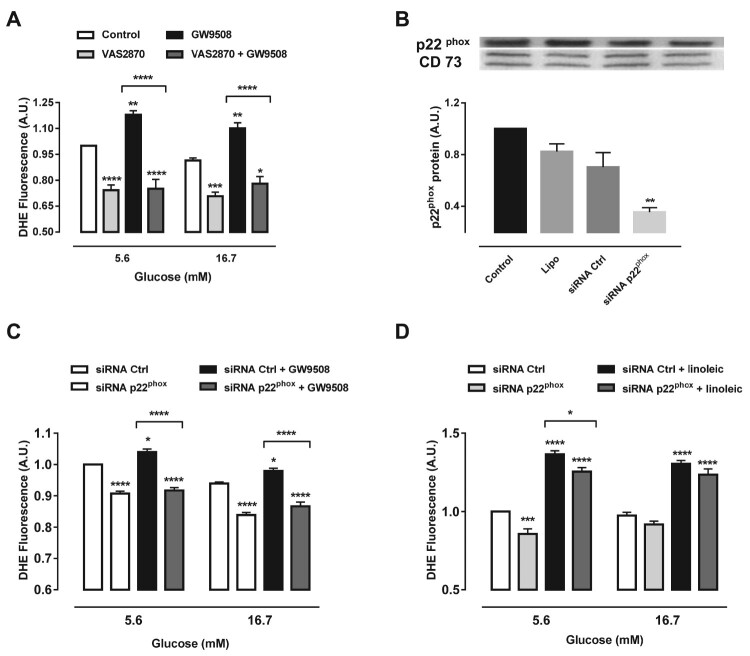
Inhibition of NADPH oxidase reduces superoxide production induced by GW9508 and linoleic acid. (A) Production of superoxide in BRIN-BD11 cells after stimulation of GPR40 with 20 μM GW9508 in the presence or absence of a NADPH oxidase inhibitor, VAS2870, at different glucose concentrations (5.6 and 16.7 mM) for 60 min. Results are expressed as mean ± SEM of 7 independent experiments. *P<0.05, **P<0.01, ***P<0.001 and ****P<0.0001 versus Control or versus GW9508 (in brackets) in corresponding glucose concentration. Two-way ANOVA followed by Tukey. (B) Protein expression of p22^phox^ in BRIN-BD11 cells submitted to small interfering RNA (siRNA) protocol. Conditions: non-transfected cells (Control), cells with lipofectamine alone (Lipo), cells transfected with scrambled siRNA (siRNA Ctrl) and cells transfected with siRNA p22^phox^ (siRNA p22^phox^). Western blot analysis was performed using anti-p22^phox^ and anti-CD73 (internal control). Results are expressed as mean ± SEM of 4 independent experiments. **P<0.01 versus siRNA Ctrl. One-way ANOVA followed by Sidak. (C) Production of superoxide in BRIN-BD11 cells transfected with scrambled siRNA (siRNA Ctrl) or siRNA p22^phox^ in absence or presence of 20 μM GW9508 in 5.6 or 16.7 mM glucose for 60 min. Results are expressed as mean ± SEM of 13 independent experiments. *P<0.05 and ****P<0.0001 versus siRNA Ctrl or siRNA Ctrl + GW9508 (in brackets) in corresponding glucose concentration. Two-way ANOVA followed by Tukey. (D) Production of superoxide in BRIN-BD11 cells transfected with scrambled siRNA (siRNA Ctrl) or siRNA p22^phox^ in absence or presence of 100 μM linoleic acid (Linoleic) in 5.6 or 16.7 mM glucose for 60 min. Results are expressed as mean ± SEM of 4 independent experiments. *P<0.05, ***P<0.001 and ****P<0.0001 versus siRNA Ctrl or siRNA Ctrl + linoleic (in brackets) in corresponding glucose concentration. Two-way ANOVA followed by Tukey. (A,C,D) Production of superoxide was analyzed by flow cytometry using DHE dye.

Next we sought to analyze the effect of NADPH oxidase on the superoxide production upon GPR40 activation. We used two approaches: (1) the pan-inhibitor of NADPH oxidase, VAS2870 and (2) siRNA of p22^phox^, which is the common membrane subunit for all NADPH oxidase isoforms. Treatment with VAS2870 led to decreased superoxide levels compared to control cells in 5.6 and 16.7 mM glucose (Figure 3(A)). In addition, VAS2870 was able to reduce superoxide levels when co-treated with GW9508 (Figure 3(A)). SiRNA for p22^phox^ resulted in a 48% reduction of the protein levels (Figure 3(B)). Furthermore, p22^phox^ knockdown prevented the increase in the superoxide content due to the activation of GPR40 by GW9508, in both glucose concentrations tested (Figure 3(C)). We have also tested whether p22^phox^ knockdown could prevent the increase in superoxide production after treatment with linoleic acid (Figure 3(D)). Indeed, siRNA for p22^phox^ was able to decrease superoxide by 8.1% when co-treated with linoleic acid in 5.6 mM glucose, with no effect in 16.7 mM glucose (Figure 3(D)).

### Effect of inhibition of NADPH oxidase on the function of β-cells after exposure to GW9508 or linoleic acid

Finally, we analyzed β-cell fitness by measuring insulin secretion and cytosolic calcium dynamics upon glucose stimulation. For functional parameters, it is well recognized that islets are better models; therefore we have used pancreatic mouse islets from Control (WT) or NOX2 KO mice.

**Figure 4.**
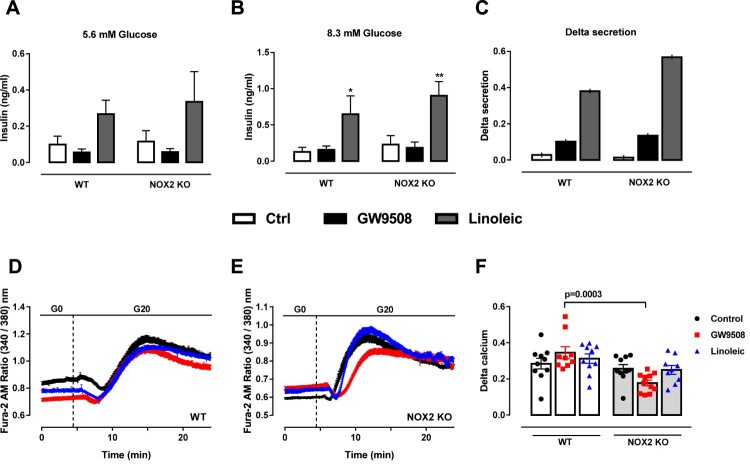
Impact of NADPH oxidase on insulin secretion and calcium dynamics induced by GW9508 and linoleic acid. (A–C) Insulin secretion of islets from C57BL/6J wild type (WT) or NOX2 deficient (NOX2 KO) mice in the absence (Control) or presence of 20 µM GW9508 (GW9508) or 100 μM linoleic acid (Linoleic) for 60 min in 5.6 mM (A) and 8.3 mM (B) of glucose. Results are expressed as mean ± SEM for 3–6 independent experiments. *P<0.05 and **P<0.01 versus Control in corresponding glucose concentration. Two-way ANOVA followed by Tukey. (C) Delta secretion (insulin secretion at 8.3 mM glucose subtracted from insulin secretion in 5.6 mM glucose). (D,E) Dynamic measurements of Ca^2+^ of islets from C57BL/6J wild type (WT) (D) or NOX2 deficient (NOX2 KO) mice (E). Islets were incubated for 60 min in the absence (Control) or presence of 20 µM GW9508 (GW9508) or 30 μM linoleic acid (Linoleic) and Ca^2+^ measurements were performed using Fura-2 AM dye under the microscope Axio Observer 7. Islets were first incubated without glucose (G0), followed by addition of 20 mM of glucose (G20). (F) Delta calcium response to glucose. Averaged values on minute 14 were subtracted from averaged values on minute 4. Results are expressed as mean ± SEM for 3 independent experiments. *P* value versus WT in same condition is shown at the graph. One-way ANOVA followed by Tukey.

We observed no difference in total insulin secretion upon GW9508 treatment for 1-hour in any glucose concentration (Figure 4(A,B)). However, when delta secretion was calculated (insulin secreted at 8.3 mM glucose subtracted by the insulin values in 5.6 mM glucose), GW9508 presented increased delta secretion compared to control group (Figure 4(C)). The lack of NADPH oxidase 2 had no impact on GW9508-induced insulin secretion in any glucose concentration tested (Figure 4(A–C)). Linoleic acid led to increased insulin secretion compared to control and GW9508 in both glucose concentrations (5.6 and 8.3 mM), and also presented higher delta secretion. However, NOX2 KO islets exposed to linoleic acid do not secrete different amounts of insulin compared to WT islets (Figure 4(A–C)).

Cytosolic calcium was measured during 1-hour incubation with GW9508 or 30 μM linoleic acid (Figure 4(D,E)). Upon stimulation with 20 mM glucose, no differences were observed in WT (Figure 4(D)) or NOX2 KO (Figure 4(E)) islets. However, NOX2 KO islets show less cytosolic calcium in GW9508, comparing with WT islets when delta calcium is calculated (average value on minute 14 minus average value on minute 4) (Figure 4(F)).

## Discussion

The potential role of NADPH oxidase on GPR40 activation and consequently on GSIS has been explored during the last few years. Activation of the NADPH oxidase complex generates ROS that may act as second messengers for GSIS [29,30,37]. In addition, GPR40 activation by FFAs or by agonist molecules also leads to the generation of additional second messengers such as DAG, Ca^2+^ and cAMP, also culminating in the enhancement of insulin secretion [1,6–8,38,39].

Our group recently demonstrated that during palmitate-induced superoxide production and stimulation of GSIS, there is a crosstalk between activation of NADPH oxidase and GPR40 in pancreatic β-cells [40]. Moreover, palmitate-induced increase in GSIS was prevented by the inhibition of NADPH oxidase (with DPI or p22^phox^ knockdown) or PKC (using calphostin) [40]. Herein, we explored whether GW9508, an agonist of GPR40, plays a role in the activation of NADPH oxidase to produce superoxide in BRIN-BD11 cells in different glucose concentrations. We also investigated whether NADPH oxidase is important for insulin secretion upon these conditions. We show that acute GPR40 activation using an agonist molecule GW9508 or linoleic acid activates NADPH oxidase and consequently increases superoxide and H_2_O_2_ levels. Interestingly, the effect was more pronounced in the presence of 5.6 and 8.3 mM of glucose, with little effect at higher glucose concentrations (16.7 mM). However, we observed no impact of deleting NADPH oxidase 2 on insulin secretion under these conditions.

The minor effects of high glucose concentration on superoxide and H_2_O_2_ are likely related to increased NADPH levels upon high glucose concentrations, as showed before [41–43]. Therefore, following glucose entry into the cell and further metabolism, NADPH is produced and used as co-factor for several ROS scavenging systems, such as glutathione reductase and thioredoxin reductase [23]. Thus, even if ROS is produced upon GPR40 activation at higher glucose concentrations, it is likely that the scavenging capacity is higher, resulting in lower net levels. In agreement, using genetically-encoded H_2_O_2_ sensors in parallel with measurements of NADPH levels, Deglasse and co-authors have shown that upon high glucose (20 mM), exogenous addition of H_2_O_2_ elicited smaller intracellular net H_2_O_2_ levels, an effect that was abolished when NADPH levels were depleted [41].

Importantly, in our study, the increase in superoxide upon GPR40 agonist was abrogated by p22^phox^ knockdown and in cells treated with a pharmacological inhibitor of NADPH oxidase (VAS2870), demonstrating that NADPH oxidases were involved (Figure 3(A,C)). Interestingly, linoleic acid, a FFA with similar binding sites to GPR40 increased superoxide levels to a higher extent than GW9508. Inhibition of NADPH oxidase by p22^phox^ knockdown led to significantly lower superoxide levels upon linoleic acid treatment; however not back to control levels. This is very reasonable, as linoleic acid is metabolized during beta-oxidation and can also lead to other sources of superoxide, such as peroxisomal and/or mitochondrial. Therefore, our data shows that while GW9508 leads to NADPH oxidase-dependent increase in superoxide levels, FFAs such as linoleic acid might increase superoxide through several mechanisms, including NADPH oxidase. In agreement, Elsner *et al*, have previously shown that upon fatty acid exposure, peroxisomes are an important source of ROS [44].

We also analyzed the effect of GW9508 on the translocation of the organizer subunit of NADPH oxidase (p47^phox^) from the cytosol to the plasma membrane at different time points (from 0 to 40 min) and different glucose concentrations. In agreement with superoxide and H_2_O_2_ measurements, p47^phox^ translocates from the cytosol to the membrane between 10 and 30 min after exposure to GW9508 in 5.6 mM, but not in 16.7 mM glucose. Migration of the p47^phox^ to the membrane, allows the assembly of the complex to produce superoxide, which is further dismutated to H_2_O_2_. Hereby we have not compared the translocation side-by-side (low vs high glucose). Therefore, it could be that upon high glucose, NADPH oxidase is already activated, as showed by others [45], and GW9508 cannot further stimulate p47^phox^ translocation. Nevertheless, the net ROS production, i.e. result between production and removal, is shifted to lower ROS levels upon acute high glucose exposure compared to low glucose.

The stimulation of GPR40 activates a Gαq protein that increases PLC activity, which in turn induces hydrolysis of phosphatidylinositol 4,5-biphosphate (PIP_2_) forming IP_3_ and DAG. DAG activates PKC which phosphorylates p47^phox^, promoting its migration to the membrane and assembly of the NADPH oxidase complex [24–26]. Previously, calphostin C, an inhibitor of PKC, was shown to prevent superoxide production after exposure of insulin-secreting cells to oleate [46] and palmitate [30].

Activation of GPR40 may enhance GSIS via Gαq by two mechanisms: IP_3_ production and oscillation in Ca^2+^ and induction of PKC/PKD activation independent of Ca^2+^ [39]. More studies are necessary to identify by which pathway GPR40 activates NADPH oxidase. Strong evidence suggests that PKC plays a key role in this crosstalk since both activations of NADPH oxidase and GPR40 involves phosphorylation by PKC [9, 24, 47–50]. However, we cannot exclude that other mechanism might also be important, such as Ca^2+^ modulation of NADPH oxidases and mitochondrial ‘ROS-induced ROS release’, where a positive feedback is established between mitochondria and NADPH oxidase, as demonstrated in other cells lines [51].

Contrary to what has been observed previously with INS-1E cells treated with palmitate [30], we did not observe a role of NADPH oxidase on insulin secretion upon GPR40 activation with GW9508 or linoleic acid. One of the main differences in these studies is that here we used mouse islets from wild type and mice knockout for NADPH oxidase 2, while in other studies, immortalized cell lines and protein knockdown or unspecific inhibitors of NADPH oxidase such as DPI were used. These inhibitors can act on different NADPH oxidases and also in other unspecific intracellular targets [52]. Nonetheless, we cannot exclude that other NADPH oxidases have impact on insulin secretion upon GPR40 activation. Interestingly, we found a significant difference in calcium oscillations upon GW9508 treatment in NOX2 KO mice islets, when stimulated with glucose (Figure 4(D–F)). One possible explanation is the fact that ROS can modulate calcium channels such as IP_3_. Indeed, studies show that exogenously added oxidants stimulate IP_3_R-mediated calcium release [53].

In conclusion, GW9508, an agonist of GPR40, acutely increases BRIN-BD11 superoxide and H_2_O_2_ content in β-cells, an effect that was prominent in lower vs higher glucose concentrations. In these conditions, GW9508 also promotes migration of the cytosolic NADPH oxidase subunit p47^phox^ to plasma membrane. Furthermore, the increase in the superoxide content after exposure to GW9508 does not occur in cells with p22^phox^ knockdown and in cells treated with the NADPH oxidase inhibitor, VAS2870. However, NADPH oxidase 2 activation upon GW9508 does not seem to be involved in insulin secretion but plays a role in cytosolic calcium levels.

We herein report for the first time a connection between the stimulation of GPR40 and the assembly of the NADPH oxidase enzymatic complex to produce superoxide and H_2_O_2_ in pancreatic β-cells, with no direct impact on insulin secretion. However, sustained superoxide and H_2_O_2_ production could have a long-term impact on β-cell fitness, leading to dysfunction upon persistent GPR40 activation. Chronic GPR40 activation have been reported to be toxic in different cell types [54–59] and clinical trials using agonist molecules had to be stopped due to cellular toxicity [60]. Whether persistent production of ROS is one mechanism deserves further attention.
